# Relationship between Sialic acid and metabolic variables in Indian type 2 diabetic patients

**DOI:** 10.1186/1476-511X-4-15

**Published:** 2005-08-10

**Authors:** Shivananda Nayak B, Geetha Bhaktha

**Affiliations:** 1 Department of Biochemistry, Kasturba Medical College, Manipal- 576104, India; 2 Department of Preclinical Sciences, Biochemistry unit, University of West Indies, Trinidad

**Keywords:** Sialic acid, Microalbuminuria, microvascular, and type-2 diabetes mellitus

## Abstract

**Background:**

Plasma sialic acid is a marker of the acute phase response. Objective is to study the relationship between sialic acid relationship with metabolic variables in Indian type 2 diabetes with and without microvascular complications.

**Research design and Methods:**

Fasting Venous blood samples were taken from 200 subjects of which 50 were of diabetes mellitus (DM) and nephropathy patients, 50 patients with type 2 diabetes and retinopathy, 50 patients with type 2 diabetes without any complications and 50 healthy individuals without diabetes. The Indian subject's aged 15–60 years with type 2 diabetes were recruited for the study. Simultaneously urine samples were also collected from each of the subjects. All the blood samples were analyzed for total cholesterol, triglyceride (TG), low-density lipoprotein (LDL), high-density lipoprotein (HDL), fasting and postprandial glucose on fully automated analyzer. Serum and urine sialic acid along with microalbumin levels were also estimated.

**Results:**

There was a significantly increasing trend of plasma and urine sialic acid with severity of nephropathy (P < 0.001) and with degree of urinary albumin excretion (P < 0.001). Serum sialic acid correlated with increasing serum creatinine concentration (P < 0.001). Elevated serum sialic acid concentrations were also associated with several risk factors for diabetic vascular disease: diabetes duration, HbA_1_c, serum triglyceride and cholesterol concentrations, waist-to-hip ratio and hypertension. Significant correlations were found between sialic acid concentration and cardiovascular risk factors like LDL and TG in the diabetic subjects.

**Conclusion:**

The main finding of this study is that elevated serum and urinary sialic acid and microalbumin concentrations were strongly related to the presence of microvascular complications like diabetic nephropathy and retinopathy and cardiovascular risk factors in Indian type 2 diabetic subjects. Further study of acute-phase response markers and mediators as indicators or predictors of diabetic microvascular complications is therefore justified.

## Introduction

Diabetes mellitus is a group of metabolic disorders characterized by elevation of blood glucose concentration and is associated with increased prevalence of microvascular complications. Type 1 diabetes mellitus results from cellular mediated autoimmune destruction of pancreatic β-cells of islets of langerhans and results in loss of insulin production. Type 2 diabetes mellitus is the most common form of diabetes accounting for 90% of cases. An estimated 16 million Americans have type-2 diabetes, and half are unaware they have it. Type 2 diabetes is characterized by insulin resistance or abnormal insulin secretion.

One of the more debilitating aspects of diabetes is the numerous complications that can arise from the disease. These complications include diabetic retinopathy, kidney nephropathy and peripheral neuropathy. The development and severity of these complications are dependent on the duration of the disease and how well it is managed. Prospective studies have reported associations among various markers of inflammation and incidence of diabetes [1], and it has been proposed that inflammation has a causal role in the development of diabetes [2]. Diabetes is another risk factor for myocardial infarction and stroke [3,4]. The relationship between diabetes and other traditional cardiovascular risk factors, e.g., an adverse lipid profile, obesity, hypertension and physical inactivity explain the increased risk in diabetic individuals. Even though it has been suggested that inflammation contributes to the increased incidence of cardiovascular diseases among diabetic subjects and only few prospective studies have addressed this question [1]. Plasma sialic acid is one of the markers for acute phase response [5]. Sialic acid is a terminal component of the non-reducing end of carbohydrate chains of glycoproteins and glycolipids [6]. Elevated total serum sialic acid (SA) concentration is a risk factor for cardiovascular mortality in humans [7]. Increased total serum sialic acid leads to increased excretion of sialic acid in urine of the patient presented with high urinary microalbumin.

It has been reported earlier that total serum sialic acid concentration increase in type 2 diabetes mellitus associated with microvascular complications [8,9]. The aim of this study was to measure serum and urine sialic acid and their relation with urinary microalbumin, serum cholesterol, TG, LDL cholesterol in diabetic subjects with and without microvascular complications.

Microalbumin is a risk factor for cardiovascular disease; it may be associated with chronic inflammation and investigated the relationship of urinary albumin excretion and urinary sialic acid.

## Materials and methods

We investigated the relationship of sialic acid concentrations with serum lipids, and urinary albumin excretion in Indian type 2 diabetic subjects of Kasturba Medical College Hospital, Manipal. The study includes 200 subjects (male and female). There were 50 healthy controls. The diabetic subjects were divided into three groups according to the level of complications. Group A-50 patients with diabetes mellitus (DM) and nephropathy, group B-50 patients with type 2 diabetes and retinopathy and group-C 50 patients with type 2 diabetes without any complications. The Indian subject's aged 15–60 years with type 2 diabetes were recruited for the study. The estimation of serum and urine sialic acid may prove to predictive and preventive of microvascular diseases and their complications in people with type 2 diabetes.

All the subjects were reported fasting in the morning after 10–12 hr overnight fast. Standing height and weight were measured. Body mass Index (BMI) defined as weight in kg/height (in meters) squared was calculated, and used as an index for obesity. Measurements of the waist circumference were taken at the mid point between umbilicus and xiphoid, and for hip circumference at the widest point around the hips. The waist hip ratio was thereafter calculated [10]. Blood pressure was measured according to the standard procedure [11].

Venous blood samples collected without the use of tourniquet from each of the patients were analyzed for total serum cholesterol, TG, LDL, HDL, fasting and postprandial glucose on fully automated analyzer (Hitachi 912 analyzer, Roche, Switzerland) with the reagents supplied by Roche. The HbA1c is estimated with the principle based on affinity chromatography technique.

Serum and urinary sialic acid was measured by a colorimetric assay using standard chemicals and reagents. In this method a protein precipitate of serum containing sialic acid will react with diphenylamine producing a purple color, which is quantitatively measured on a spectrophotometer at 540 nm.

The fresh urine samples collected from the test and control group subjects were used for microalbumin estimation in an electrochemiluminiscence analyzer (Roche, Switzerland).

### Statistical Method

Results were expressed as mean ± S.D. except where otherwise stated. Data were analyzed using the statistical package for social science, SPSS and p value ≤ 0.05 was taken as the cut off level for significance. Because the distribution of most variables was not symmetric. We used non-parametric statistical methods. Chi square tests was be used to examine, type 2 diabetes mellitus, the various clinical and biochemical markers.

## Results

The table 1 shows the relationship between serum sialic acid, urine sialic acid and microalbumin concentrations with metabolic variables in diabetic subjects with and without microvascular complications. The table depicts significant increase of serum sialic acid (< 0.001) among the Indian diabetic subjects compared to the control subjects. Furthermore, in the diabetic subjects urine sialic acid and microalbumin were significantly higher (< 0.001). The Table also shows the association of sialic acid and several risk factors for diabetic vascular disease; diabetes duration, serum TG and cholesterol concentration. It is observed that the sialic acid values were statistically significantly higher with increasing urinary albumin excretion (p < 0.001). Similarly HbA_1_c, FBS, PPBS, TG and cholesterol showed marked increase in patients with elevated level of microalbumin and urine sialic acid when compared to normal subjects without any complications.

**Table 1:** Serum and urinary sialic acid and microalbumin levels in Type 2 diabetes with nephropathy and retinopathy

Parameters	Diabetes without any complications	Diabetic nephropathy	Diabetic Retinopathy	Non-diabetic subjects	p value
Serum Sialic acid (mg%)	55.05 ± 2.9*	85.05 ± 2.7***	75.05 ± 2.5***	46.6 ± 2.08	< 0.001
Urine sialic acid (mg%)	6.02 ± 2.58**	13.06 ± 1.58***	11.03 ± 1.78***	3.2 ± 0.65	< 0.001
Microalbumin (mg %)	8.2 ± 3.24	132.2 ± 35.24***	102.2 ± 29.24***	7.67 ± 3.28	< 0.001
FBS (mg%)	140.02 ± 70.08	155.6 ± 50.7	150.6 ± 49.9	90.02 ± 80.08	< 0.01
PPBS (mg%)	150.02 ± 102.10	207.3 ± 57.6	200.1 ± 57.6	120.02 ± 102.10	< 0.01
HbA_1C _(%)	9.10 ± 5.20	11.1 ± 2.3	10.1 ± 2.5	6.10 ± 5.20	< 0.05
Triglyceride (mg%)	122.04 ± 75.01	178.02 ± 78.01	180.04 ± 75.01	120.04 ± 76.01	< 0.05
Cholesterol(mg%)	148.04 ± 120.01	256.03 ± 134.01	246.03 ± 130.01	140.04 ± 119.01	NS
HDL (mg %)	35.01 ± 20.04	38.01 ± 26.02	35.01 ± 20.04	36.01 ± 19.04	NS
LDL (mg %)	90.00 ± 76.06	165.00 ± 97.01	160.00 ± 95.01	87.00 ± 76.05	< 0.05
Creatinine (mg %)	2.00 ± 1.6.06	10.05 ± 2.03	10.03 ± 2.01	1.40 ± 1.20	< 0.001
Urine creatinine (mg%)	145.00 ± 102.6	155.03 ± 65.02	150.03 ± 66.01	146.00 ± 113.06	< 0.05

Mean ± SD *** p < 0.001, NS = not significant, n = 50

## Discussion

In recent years, much attention has been given to the relationships among adiposity, inflammation, and diabetes. High inflammation sensitive plasma protein levels increased the cardiovascular risk slightly more in diabetic. Studies of diabetic subjects have reported increased incidences of cardiovascular diseases or increased diabetes complications among subjects with high fibrinogen [12] and other markers of inflammation [13,14]. Measurement of inflammation sensitive markers may be useful for assessment of the cardiovascular risk in diabetic patients. Results from prospective studies suggest that inflammation involved in the pathogenesis of diabetes [15] and atherosclerosis [16]. Inflammation could be a common antecedent for both diabetes and cardiovascular disease. Hyperglycemia and insulin resistance could also promote inflammation, and may be factor linking diabetes to the development of atherosclerosis. Elevated glucose levels could promote inflammation by increased oxidative stress [17]. Yet another possibility is that the inflammatory response is a result of vascular complications following diabetes. In type-2 diabetes, the circulating sialic acid concentration is elevated in comparison with non-diabetic subjects [18]. The results of our study showed serum and urine SA concentration increased in diabetic patients as compared to the general population, especially in type-2 diabetic patients with either microalbuminuria or albuminuria. Furthermore, the serum and urine sialic acid levels were independent of the duration of diabetes mellitus and degree of metabolic control (as estimated by HbA_1_c). Also, a good correlation was observed between sialic acid and important cardiovascular risk factors such as cholesterol, LDL and TG.

It has been reported that serum sialic acid levels are increased in type1 DM patients with albuminuria [19]. Several authors found the increased urinary concentration of sialic acid in type 2 diabetes with microangiopathy. The vascular permeability is regulated by sialic acid moieties, with increased vascular permeability resulting from the shedding of vascular endothelial sialic acid into the circulation. It is well established that vascular endothelium carries a high level of sialic acid [20], and the vascular damage leads to its release into the circulation. A relationship between serum sialic acid levels and microvascular complications has been observed before for microalbuminuria and clinical proteinuria in type 1 [21] and type 2 diabetes [22].

Our findings clearly indicated that serum and urinary sialic acid concentrations were elevated in type-2 diabetes. Crook M *et.al *found that serum sialic acid was significantly higher in men with diabetic complications than in those without any of the complications [23]. There may be an association between sialic acid and complications through the acute phase response.
